# Development of an IgY-Based Treatment to Control Bovine Coronavirus Diarrhea in Dairy Calves

**DOI:** 10.3390/v15030708

**Published:** 2023-03-09

**Authors:** Marina Bok, Celina G. Vega, Matias Castells, Rodney Colina, Andrés Wigdorovitz, Viviana Parreño

**Affiliations:** 1 INCUINTA, Virology Institute and Technology Innovations, National Institute of Agricultural Technology, Buenos Aires 1686, Argentina; 2 CONICET, Buenos Aires C1425FQB, Argentina; 3 Bioinnovo S.A., Buenos Aires 1686, Argentina; 4 Molecular Virology Laboratory, CENUR North Litoral, Salto University Centre, University of the Republic, Salto 50000, Uruguay; 5 Animal Health Research, National Institute of Agricultural Research (INIA), Colonia 70006, Uruguay

**Keywords:** neonatal calf diarrhea, bovine coronavirus, IgY antibodies, development, ELISA validation

## Abstract

Bovine Coronavirus (BCoV) is a major pathogen associated with neonatal calf diarrhea. Standard practice dictates that to prevent BCoV diarrhea, dams should be immunized in the last stage of pregnancy to increase BCoV-specific antibody (Ab) titers in serum and colostrum. For the prevention to be effective, calves need to suck maternal colostrum within the first six to twelve hours of life before gut closure to ensure a good level of passive immunity. The high rate of maternal Ab transfer failure resulting from this process posed the need to develop alternative local passive immunity strategies to strengthen the prevention and treatment of BCoV diarrhea. Immunoglobulin Y technology represents a promising tool to address this gap. In this study, 200 laying hens were immunized with BCoV to obtain spray-dried egg powder enriched in specific IgY Abs to BCoV on a large production scale. To ensure batch-to-batch product consistency, a potency assay was statistically validated. With a sample size of 241, the BCoV-specific IgY ELISA showed a sensitivity and specificity of 97.7% and 98.2%, respectively. ELISA IgY Abs to BCoV correlated with virus-neutralizing Ab titers (Pearson correlation, R^2^ = 0.92, *p* < 0.001). Most importantly, a pilot efficacy study in newborn calves showed a significant delay and shorter duration of BCoV-associated diarrhea and shedding in IgY-treated colostrum-deprived calves. Calves were treated with milk supplemented with egg powder (final IgY Ab titer to BCoV ELISA = 512; VN = 32) for 14 days as a passive treatment before a challenge with BCoV and were compared to calves fed milk with no supplementation. This is the first study with proof of efficacy of a product based on egg powder manufactured at a scale that successfully prevents BCoV-associated neonatal calf diarrhea.

## 1. Introduction

Neonatal calf diarrhea (NCD) is a multifactorial disease in newborn calves on farms worldwide [1]. Bovine coronavirus (BCoV) associated with NCD has been detected in beef and dairy herds on all continents causing large economic losses to the dairy industry [2,3]. This enteric disease affects calves between 24 h and 30 days of life, although cases as late as 5 months of age have been reported [4,5,6]. The peak incidence occurs between days 7 and 10 [5,7,8]. The strains detected in calves with diarrhea have been labeled as BCoV-induced calf diarrhea strains (BCoV-CD) and belong to the bovine enteric or enteropathogenic coronaviruses (BECoV) group.

Worldwide, BCoV prevalence varies between 15 and 70% in naturally occurring outbreaks [9,10,11], but antibody (Ab)-prevalence is over 90% in adult cattle, suggesting that most of the cattle are exposed to the virus at least once in their lifetime [2]. However, the rate of fecal detection of BCoV ranges from 5.95% [12] to 10.52% [7] in herds from Argentina and is at 7.8% in herds from Uruguay [5]. Studies show that BCoV infection rates are significantly higher in dairy than in beef herds in Argentina, while the prevalence is higher in beef herds in Uruguay [5].

BCoV belongs to the Betacoronavirus genus within the *Coronaviridae* family. Infection is mainly induced by fecal-oral and respiratory transmission routes with varying viral tropisms depending on the BCoV spike protein [13]. Calves become infected early after birth through fecal exposure from carrier dams. BCoV diarrhea is detected mainly in artificially reared dairy calves. Once infected, a calf can shed high amounts of the virus (e.g., 1 × 10^9^ virus particles per mL of feces). The pathology of BCoV is more severe than that of bovine group A rotavirus (RVA), resulting in mucohemorrhagic enterocolitis [14]. BCoV causes severe diarrhea, sometimes fatal, in young animals destroying epithelial cells from the gut’s intestinal villi, causing malabsorption syndrome [15]. Virus replication leads to the destruction of the absorptive intestinal villous epithelial cells in the entire small intestine and eventually spreads throughout the large intestine, causing atrophy of colonic ridges and malabsorptive diarrhea. Undigested nutrients are fermented in the large intestine, promoting bacterial overgrowth and production of organic acids, especially D-lactate, contributing to electrolyte loss and leading to dehydration and metabolic acidosis. As a rule, the severity of the disease increases while the incubation period decreases in younger calves, especially those with failure of passive immune transfer (FPT). The majority of calves recover, but a few may experience pyrexia, recumb, cardiovascular collapse, coma, and death [4,16].

BCoV is also associated with winter dysentery in adult cattle [17] and respiratory tract disorders in cattle of all ages [18,19]. Some BCoV strains behave as pneumoenteric pathogens, also replicating in the upper respiratory tract. Infected calves may develop both diarrhea and respiratory disease, shedding the virus in feces and nasal secretions [4,16].

Calves acquire passive maternal protective Abs against common pathogens via colostrum intake. For a successful passive transfer from mother to calf, the calf needs to suck maternal colostrum within the first six to twelve hours of life before gut closure, so that maternal Abs and other macromolecules can be transferred massively into the bloodstream [20]. Systemic maternal Abs are then transferred back to the gut lumen by active transport via the neonatal Fc receptor [21,22].

Although dams may be vaccinated against BCoV to control diarrhea in calves, the frequency of FPT poses the need to develop alternative local passive immune strategies to enhance prevention and, most importantly, as a specific treatment method to control BCoV diarrhea in infected calves. Milk supplements studies have been conducted to prevent diarrhea caused by other viruses such as RVA. Prevention and treatment are based on specific Abs from colostrum (homologous IgG) [23] and egg yolk Abs (heterologous IgY) as passive immune therapies [24,25,26]. Moreover, IgY Abs successfully treated SARS-CoV-2 infection when given intranasally to animals and humans [27,28].

IgY Abs transference from chicken serum to the egg after immunization was initially described in 1893 [29]. IgY Abs are the only Ab isotype present in chicken egg yolk, simplifying processing and purification, and approximately 1500 mg of IgY can be harvested each month from each laying hen (5–25 mg/egg yolk), while 2 to 10% of total Ab are antigen-specific IgY [30]. These properties make IgY production a fast and economic method for polyclonal Ab production [31,32]. It is also advantageous to use avian IgY instead of mammalian IgG based on animal welfare [30]. Our studies have previously described that prophylactic or therapeutic administration of hyperimmune egg yolk to newborn calves during the first days of life significantly reduced the severity of diarrhea after a challenge with virulent bovine RVA [25,26]. In contrast, there are just a few studies on BCoV prevention strategies for bovine and porcine species using IgY Abs [33,34,35]. There is only one study that reported that the administration of IgY Abs against BCoV in calves was efficacious against diarrhea [36].

Additionally, every new biological measure needs to be developed together with standardized manufacturing methods and suitable laboratory techniques for chemistry manufacturing and control (CMC) standards that adhere to regulatory authorities’ requirements. This study also describes the technology used to achieve this goal. The specific aims of this study are (i) to produce on a large-scale spray-dried egg yolk powder enriched in IgY Abs against BCoV; (ii) to evaluate batch-to-batch consistency using a statistically validated potency assay; and (iii) to conduct a proof of efficacy study where IgY to BCoV powder was administered as a prophylactic treatment to colostrum-deprived calves experimentally infected with BCoV vs. animals fed milk without IgY supplementation.

## 2. Materials and Methods

### 2.1. Egg Powder Enriched with IgY Abs to BCoV

Batches of egg powder used in this study were obtained from BCoV hyperimmunized hens. Two hundred White Leghorn laying hens were housed in groups of eight animals per cage at the laying hen facility of Bioinnovo S.A., Buenos Aires, Argentina. Room temperature, relative humidity, and light/dark cycles were controlled. Hens were fed a laying hen diet and water ad libitum, and eggs were collected daily starting at the first immunization. The vaccine contained 10^8^ Fluorescence Focus Forming Units (FFU)/mL of live attenuated BCoV (Mebus reference strain) mixed in a 1:1 ratio with ISA 71VG adjuvant (Seppic, Courbevoie, France). For the emulsification process, oil and water components were mixed using a T25 digital Ultraturrax (IKA, Baden-Württemberg, Germany). This strain was selected following regulations from the Argentinean animal health service authority (SENASA), which requested that future batches of BCoV IgY be produced with commercially approved BCoV vaccines since this development is planned to be scaled up to register a commercial product. Commercial BCoV vaccines licensed in Argentina are designed for bovines and formulated based on the Mebus strain. Thus, in anticipation of eventually immunizing with a commercial BCoV vaccine, the hen vaccine was formulated with the same strain, the Mebus strain. However, the treated and untreated calves were subsequently challenged with the Arg95 BCoV strain during the efficacy study to evaluate the cross-protection to the strain currently circulating in Argentina.

Hens were immunized intramuscularly in the breasts with 0.5 mL of the immunogen at 0, 15, 30, 90, 120, 180, and 195 days. BCoV-specific IgY Abs were measured in sera from ten randomly selected hens to track the elicited immune response to the immunogen. Additional booster doses were administered when a decrease in the IgY Ab titer was detected in order to maintain high BCoV-specific IgY titers throughout the study.

To obtain egg powder batches, whole eggs from immunized hens were processed using an egg breaker (Pelbo, Brugherio, Italy) and then spray-dried (FlexPump, Galaxie S.A., Buenos Aires, Argentina). IgY Ab titers were determined by ELISA. Batches with the highest BCoV IgY Ab titers were tested by virus neutralization (VN) assay and maintained at a controlled room temperature (18–24 °C) until use. The product quality and stability were studied over 2 years and the physicochemical and microbiological properties were evaluated as previously reported [26].

### 2.2. BCoV IgY Specific Antibody ELISA

IgY Ab titers to BCoV were measured in samples of egg powder, calf sera, calf feces, and supplemented milk using a double-antibody sandwich ELISA. Briefly, 96 well ELISA plates (Maxisorp, NUNC, Thermo Fisher, Waltham, MA, USA) were coated with a BCoV hyperimmune antiserum (ELISA BCoV-IgG titer of 16384) prepared in guinea pigs at a 1:5000 dilution in carbonate/bicarbonate buffer pH 9.6. After a blocking step with 10% non-fat milk (La Serenísima, Mastellone Hnos, Buenos Aires, Argentina), the supernatant of cell culture lysate BCoV-infected or mock-infected HRT-18 cells was added, followed by serial four-fold dilutions of the samples. The reaction was developed using a peroxidase-labeled polyclonal goat IgG anti-chicken IgY Ab (Sigma-Aldrich, Burlington, MA, USA) as conjugate and hydrogen peroxide and ABTS (2,2′-Azino-bis (3-ethylbenz-thiazoline-6-sulfonate)) as substrate/chromogen system. This assay was statistically validated to enable a robust tool for quality control of drug substance lots. For this objective, a positive and negative reference sample population (chicken serum and egg yolk) was obtained from BCoV-immunized and non-immunized hens, respectively (*n* = 241 samples). Furthermore, parameters of analytic and diagnostic validation were assessed following WOAH recommendations [37].

### 2.3. Fluorescent Focus Reduction virus Neutralization Test

Virus-neutralizing Ab titers to Mebus and Arg95 BCoV in hens’ sera and egg yolk pools, supplemented milk, and calf sera were measured with a fluorescent VN test as previously described [12]. Briefly, serial 4-fold dilutions from samples were pre-incubated with 100 TCID of BCoV Mebus or Arg95 strain for 1 h at 37 °C. Sample and virus mixtures were added to HRT-18 cell monolayers and incubated for 48 h at 37 °C and 5% CO_2_. Finally, monolayers were fixed with 70% acetone and incubated with an anti-BCoV guinea pig serum FITC-labeled (1:100 in 0.1% in PBS Evan’s blue solution) for 1 h at 37 °C. Fluorescence foci (representing the infected cells) were observed under a fluorescence microscope. The Ab VN titer was expressed as the reciprocal of the highest sample dilution that resulted in a >80% reduction in the number of fluorescent foci.

### 2.4. Virus and Inoculum Production

BCoV Mebus and Arg95 strains were replicated in HRT-18 cells in D-MEM (Gibco, Thermo Fisher, Waltham, MA, USA) plus 2 µg/mL of bovine trypsin (Sigma-Aldrich, Burlington, MA, USA) and antibiotics during 48–72 h at 37 °C and 5% CO_2_ atmosphere. After observation of the cytopathic effect, cells and virus were frozen and thawed followed by a clarification step of 3500 rpm for 20 min. Supernatants containing viral strains were stored at −80 °C until use.

The challenge stock was obtained from one seven-day-old colostrum-deprived calf that had been infected orally with 50 mL of the BCoV Arg95 strain (10^5^ FFU/mL). Once BCoV shedding was confirmed, 72 h after virus inoculation, the calf was euthanized and different aliquots of intestinal content were titrated by antigen ELISA, cell culture immunofluorescence assay (CCIF), and RT-qPCR. To confirm the infectivity of the challenge stock, a second calf was inoculated with 20 mL of the first calf’s fecal fraction diluted in D-MEM with antibiotics and filtered by a 0.2 µm syringe filter. Microbiological controls and back titration of the inoculum were performed to confirm its identity. Twenty mL of the fecal fraction containing 10^6^ FFU of BCoV were used to challenge calves in the efficacy study.

### 2.5. Experimental Design to Test the Efficacy of Egg Yolk Powder to Prevent BCoV Diarrhea in Calves

**Figure 1:**
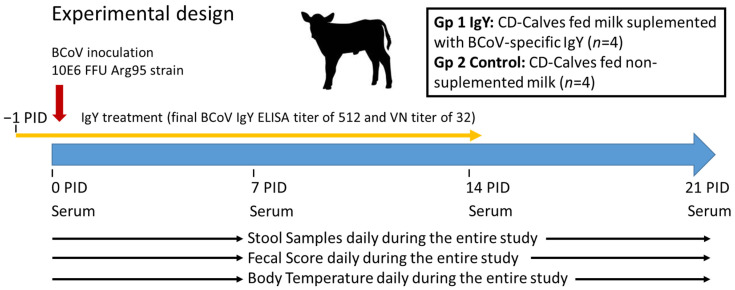
Experimental design of efficacy study. Gp = group; PID = Post Inoculation Day.

The experimental design to test the efficacy of egg powder against BCoV is summarized in Figure 1. Eight newborn Holstein male calves procured from the same dairy farm were separated from their mothers at birth before suckling and moved into the isolation facilities within the first 4 h of life. The animals were housed in individual isolation rooms under a strict management protocol as previously described [38]. All calves were confirmed to be negative for BCoV-specific IgG by ELISA at 48 h of life. All animals received 2 L of Ab-free milk twice a day as regular feeding. The milk used in the experiment was commercially sterilized 3% fat bovine milk apt for human consumption (La Serenísima, Mastellone Hnos, Buenos Aires, Argentina). Calves were randomly assigned to one of the two following experimental feeding groups: Gp 1 = calves fed with milk supplemented with 40 g of BCoV-specific egg powder with a final ELISA BCoV IgY Ab titer of 512 and VN of 32, twice a day (*n* = 4); Gp 2 = calves fed milk without supplementation (*n* = 4). IgY Ab titers in milk were determined by ELISA and VN. All animals were inoculated orally with 10^6^ FFU of virulent BCoV Arg95 strain approximately one week after birth [0 post-inoculation day (PID)]. This viral dose was previously confirmed to cause diarrhea and virus shedding in 100% of inoculated colostrum-deprived calves (control calves). Animals assigned to the IgY passive treatment group (Gp 1) started the treatment 24 h before the virus inoculation day (−1PID). From PID 15 onwards, all calves in both groups were fed with milk without supplemental Abs (end of treatment window).

Calves were examined daily for signs of diarrhea and virus shedding after BCoV inoculation. The presence of elevated rectal temperatures was recorded. To estimate the severity of diarrhea, fecal consistency was scored as follows: 0: normal; 1: pasty; 2: semi-liquid; and 3: liquid. A score equal to or greater than 2 was considered diarrhea. The scoring was done blindly by qualified technicians. Before and after BCoV inoculation, fecal samples were collected daily to assess virus shedding. Serum samples were collected on the day of birth, at inoculation, and then weekly (7, 14, 21 PID). Serum Abs to BCoV were evaluated by ELISA and VN assays. The presence of Abs in feces was assessed by ELISA.

### 2.6. Coronavirus Antigen Detection

BCoV shedding was detected in fecal samples using an antigen capture ELISA as described previously [39]. The genotype and molecular identity of BCoV were confirmed by RT-PCR followed by DNA sequence analysis as described elsewhere [12].

### 2.7. Quantitative Real-Time BCoV PCR

Viral RNA was extracted using QIAamp^®^ Viral RNA Mini Kit (Qiagen, Germantown, MD, USA), following the manufacturer’s instructions. Reverse transcription (RT) was carried out with MML-V (Promega, Madison, WI, USA) and random hexamers primers (Thermo Fisher, Waltham, MA, USA). Five microliters of RNA were used as a template and cDNA was stored at −20 °C. Quantitative polymerase chain reactions (qPCR) were performed with TaqMan^®^ technology on an ABI 7500 instrument (Applied Biosystems, Thermo Fisher, Waltham, MA, USA); cDNA from fecal samples was analyzed in duplicate. To quantify BCoV in the samples, a standard curve was generated by using ten-fold dilutions (10^7^ to 10^2^ FFU/mL) of a tissue-cultured Mebus BCoV strain. A fragment of N gene, which is highly conserved among strains, was amplified. As was described elsewhere, five microliters of cDNA were used as the template, 2× MasterMix™ (Applied Biosystems, Thermo Fisher, Waltham, MA, USA), and primers and probes with a final concentration of 0.4 μM and 0.2 μM, respectively, were also used [40]. DNase/RNase-free water to a final volume of 25 μL completed the reaction mix.

### 2.8. Cell Culture Immunofluorescence Assay (CCIF)

The virus infectious titer was assessed with a cell culture immunofluorescence (CCIF) assay [12]. Fluorescent foci within the infected well were visualized using a FITC-labeled hyperimmune guinea pig antiserum to BCoV, and fluorescent foci-forming cells were counted using a fluorescence microscope. Titers were expressed as the number of FFU/mL and log-transformed for area under the curve (AUC) calculation.

### 2.9. Bovine Isotype-Specific Antibody ELISA

The IgM, IgA, and IgG1 Ab titers to BCoV were quantitated in calf sera and feces. Specific IgG1 Abs were detected by a double-sandwich ELISA using the reagents and protocol described previously [8]. Specific IgM and IgA Abs were measured by capture-ELISAs standardized in our laboratory following previous studies [38] and using anti-bovine IgM mAb (kindly supplied by Dr. L.J. Saif, The Ohio State University, Wooster, OH, USA) and affinity-purified sheep anti-bovine IgA polyclonal antibody (Bethyl Laboratories INC., Montgomery, TX, USA). Briefly, 96-well plates (Maxisorp, NUNC, Thermo Fisher, Waltham, MA, USA) were coated overnight at 4 °C with anti-IgA and anti-IgM Abs in a dilution 1/500 in a carbonate-bicarbonate buffer (pH 9.6) and blocked with 10% non-fat milk (La Serenísima, Mastellone Hnos, Buenos Aires, Argentina) in PBS. Plates were washed four times with 0.5% Tween 20 in PBS, pH 7.4, before adding 100 µL of the four-fold dilution of each sample per well and incubating for 1 h at 37 °C. Clarified Mebus BCoV and mock-infected HRT-18 cell supernatants were added in volumes of 100 µL/well. As detector Ab, a hyperimmune guinea pig serum to BCoV was added at a dilution of 1/5000 and incubated for 1 h at 37 °C. Finally, the reaction was developed using a commercial HRP-conjugated anti-guinea pig IgG Ab in a dilution of 1/5000 (Seracare, Milford, MA, USA) and hydrogen peroxide and ABTS as substrate/chromogen system. The reaction was stopped with 50 µL of 5% sodium dodecyl sulfate (SDS) (Sigma-Aldrich, Burlington, MA, USA). The absorbance at 405 nm was measured using an ELISA plate reader (Thermo, Marsiling Industrial Estate, Singapore). The ELISA Ab titer of each sample was expressed as the reciprocal of the highest dilution that had a corrected absorbance value (absorbance in the virus-coated well minus absorbance in the mock antigen-coated well for each sample and dilution) greater than the cut-off value (mean corrected absorbance of positive capture of blank wells + 3 standard deviations (SD)). Samples negative at a dilution of 1:4 were assigned a titer of 2 for the calculation of geometric mean titers (GMTs).

### 2.10. Statistical Analysis

The cut-off value of the ELISA assay to measure anti-BCoV IgY Abs was obtained after performing the Receiver Operating Curve (ROC) analysis of the corrected optical densities (ODc) measured at 405 nm. The ODc frequency distribution from 241 samples classified 94 as negative and 147 as positive, tested at 1/256 dilution (reference population). Diagnostic sensitivity and specificity of the IgY ELISA were estimated following the Greiner and Gardner recommendations and validation parameters of The WOAH Manual of terrestrial animals [41]. Concordance analysis between the classification of reference samples and its results obtained by the ELISA was conducted using the Kappa statistic [42]. To estimate the intermediate precision of the ELISA, ODc results from the positive control were recorded for a period of four years (2012–2015). The result was expressed as the coefficient of variation (CV). The correlation between log-transformed VN and ELISA IgY Ab titer was analyzed by linear regression and Pearson’s correlation test. Statistical analyses were conducted using MedCalc Software (Ostend, Belgium), Version 19.0.5, and Infostat (Córdoba, Argentina), Version 2020.

Statistical analysis of the efficacy study in calves was conducted as follows. Cumulative titers of virus shedding were expressed as AUC. The AUC of diarrhea score and virus shedding was calculated in RStudio (Boston, MA, USA) using the AUC command of the DescTools package and the spline approach. The mean day of onset, duration of virus shedding and diarrhea, and mean AUC of virus shedding and diarrhea among the treatment groups were compared using the Student *t* test or Wilcoxon rank sum test when the normality and homoscedasticity assumptions were not met. Neutralizing and isotype-specific Ab titers in feces and serum were log10-transformed and serum negative samples at a dilution of 1:2 were assigned an arbitrary Ab titer of 2, log = 0.3, for the calculation of geometric mean titers (GMTs) in the case of the inverse of the maximum positive dilutions and arithmetic mean in the case of the log-transformed values. The IgM, IgA, and IgG antibody titers in serum from PID 0 to PID 14 and the virus shedding titer and diarrhea score from PID 1 to 14 were analyzed by a two-way ANOVA of repeated measures through time. Statistical significance was assessed at *p* < 0.05. Statistical analyses were performed using Infostat with a link to R or RStudio Version 1.4.1717 from RStudio, PBC.

## 3. Results

### 3.1. Development of a Product Based on IgY Abs to BCoV

**Figure 2:**
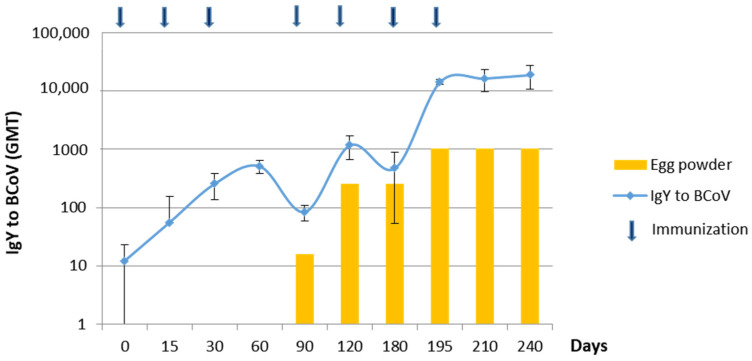
Laying hens immunization and IgY antibody (Ab) responses to bovine coronavirus (BCoV). Geometric Mean Titers (GMT) (with error bars) of serum BCoV-specific IgY titers from 10 hens randomly sampled at each time point. Geometric Mean Titers (GMT) of IgY Abs to BCoV in spray-dried egg batches (columns) determined by ELISA. Arrows indicate immunization times.

Laying hens were immunized with a vaccine formulated with the BCoV Mebus reference strain in an avian-specific adjuvant as described in the M&M section. This vaccine was highly immunogenic in hens reaching ELISA IgY Ab titers to BCoV in the serum of 18,820 (GMT). The egg powder batches reached a peak of ELISA IgY Ab titer to BCoV of 1024 measured in a suspension of 10 mg/mL (Figure 2). Eggs from 200 hyperimmunized hens were collected daily and were crashed and dried every two weeks at the Bioinnovo manufacturing plant generating two DS (Drug Substance) batches with a total of 74.2 kg of dried egg-based products. IgY Ab titers from all DS batches are detailed in Table 1. Additionally, we evaluated the immune response against several antigens, independently, when we formulated polyvalent vaccines for hens, and we observed no negative effect (decrease in overall Ab titer) in either IgY to BCoV response or IgY response to other antigens as RVA when both antigens were formulated together.

### 3.2. Quality Control of Egg Yolk Powder DS Batches Enriched in IgY to BCoV

IgY Ab neutralizing activity against Mebus and Arg95 BCoV strains and binding IgY Ab titers in egg powder batches and supplemented milk are shown in Table 1. VN Ab titers against the Mebus and Arg95 strains were equal. As expected, VN Ab titers were lower than binding IgY titers in the produced batches (64 vs. 1024, respectively) as well as in the supplemented milk (32 vs. 512 respectively). This result predicted that IgY Abs might serve as a potential tool to neutralize BCoV infection when administered in vivo.

### 3.3. BCoV IgY-Specific Antibody ELISA Validation

A summary of all diagnostic validation results is depicted in Table 2. The assay was successfully validated according to WOAH recommendations, giving assay specificity and sensitivity values of 98.2% and 97.7%, respectively.

#### 3.3.1. Positive Control: Admission Range and Linearity

**Figure 3:**
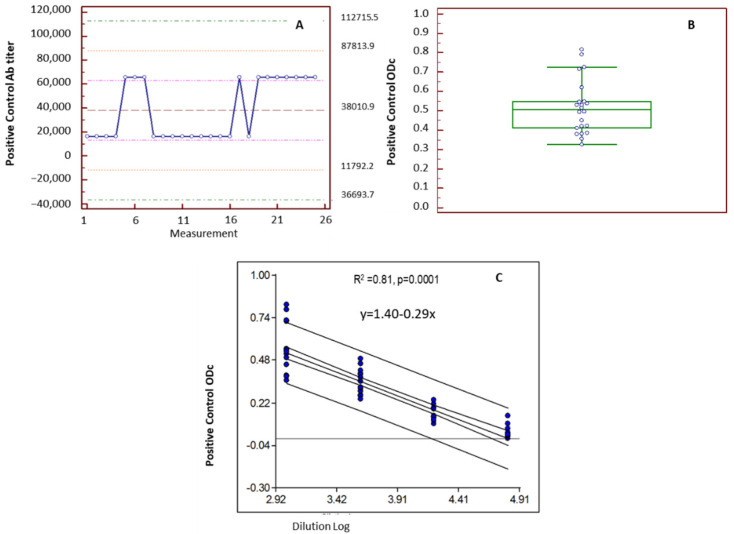
(**A**) Control Chart of the positive control of the ELISA assay. Ab titers obtained in 25 different plates run in 13 assays for 4 years (2012–2015). (**B**) Box and Whisker plot showing the admission range of the positive control. (**C**) Linear regression analysis of positive control to establish the linearity range of the assay.

A pool of sera from four hens immunized with BCoV was used to produce the positive control of the assay. The sera pool had an IgY Ab titer to BCoV that ranged between 16,384 and 65,536, resulting in a mean expected titer of 32,768 (95% CI: 22,564–40,293). These titers were obtained after running four-fold serial dilutions of the positive control in 25 plates of ELISAs run from 2012 to 2015 (Figure 3A).

To determine the admission range of the positive control, samples were run 22 times in a 1/1024 working dilution resulting in an ODc of 0.518 (0.378–0.658; arithmetic mean ± 1 SD). For an individual assay result to be characterized within the expected parameters, the ODc of the positive control had to fall within this admission range, while the negative control and the blank had to measure below the cut-off of the assay (Figure 3B).

The linearity of the positive control was determined by running serial four-fold dilutions of the positive control in eight independent assays during four consecutive years. A linear decrease of ODc vs. dilution factor of the positive control allowed for adjusting the linear regression in the dilution range tested (Figure 3C).

#### 3.3.2. ROC Curve: Cut-Off Value and Associated Sensitivity and Specificity

**Figure 4:**
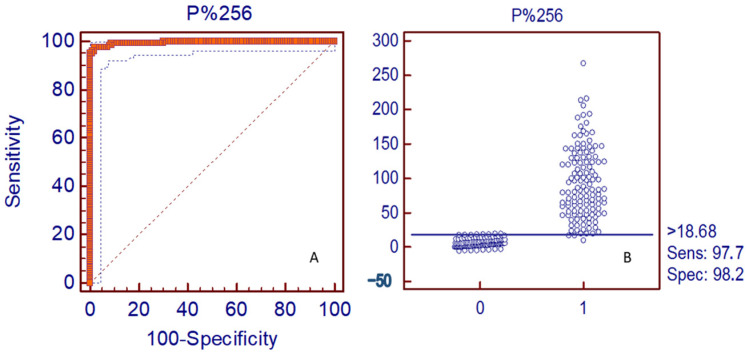
(**A**) Receiver Operating Curve (ROC) of samples at 1/256 dilution. (**B**) Reference population classification using the suggested cut-off value of 18.68 P%. Numbers 0 and 1 represent negative and positive samples, respectively. Results were expressed as the percentage of positivity regarding the ODc of the positive control run in each plate.

The cut-off value of the assay was determined after ROC analysis on the frequency distribution of ELISA ODc from 241 samples. A total of 94 samples were classified as negatives (serum, fluid egg yolk, and dried egg powder from non-immunized hens or hens immunized with other antigens including Rotavirus, *Salmonella*, and *E. coli*) and 147 were classified as positive (serum, egg yolk, and dried egg powder from BCoV immunized hens) constituting the reference population. Samples were tested by ELISA in 1/256 dilution (Figure 4B). The 1/256 dilution was selected for the validation analysis because this was the minimum dilution that showed significant differences between the OD obtained in virus-coated and mock-coated wells.

Results were expressed in percentage of positivity (P%) from the ODc of the positive control run in each plate in a 1/1024 dilution which was assumed as 100 P%. The ROC analysis indicated that at a cut-off value of 18.68% from the ODc of positive control (tested in each plate in a 1/1024 dilution), the sample to be tested will be properly classified 95 out of 100 times. With this 18.68 P% cut-off value, the assay showed a diagnosis sensitivity (DSe) and specificity (DSp) of 97.66% and 98.23%, respectively (Figure 4B). The assay could detect the two different positive and negative populations independently of the sample dilution used. The cut-off value measured in ODc was 0.110.

A concordance analysis between IgY to BCoV ELISA results and the actual classification of the samples included in the reference population considering an 18.68 P% cut-off value showed a Kappa value of 0.93 corresponding to an almost perfect agreement (Table 3).

#### 3.3.3. Assay Intermediate Precision

The CV for the IgY to BCoV ELISA resulted in 27%, which is less than the 30% admitted by WOAH [37]. This analysis included intra-plate repeatability and reproducibility in separate assays.

#### 3.3.4. Correlation between the IgY ELISA Ab Titers and Neutralizing Ab Titers

**Figure 5:**
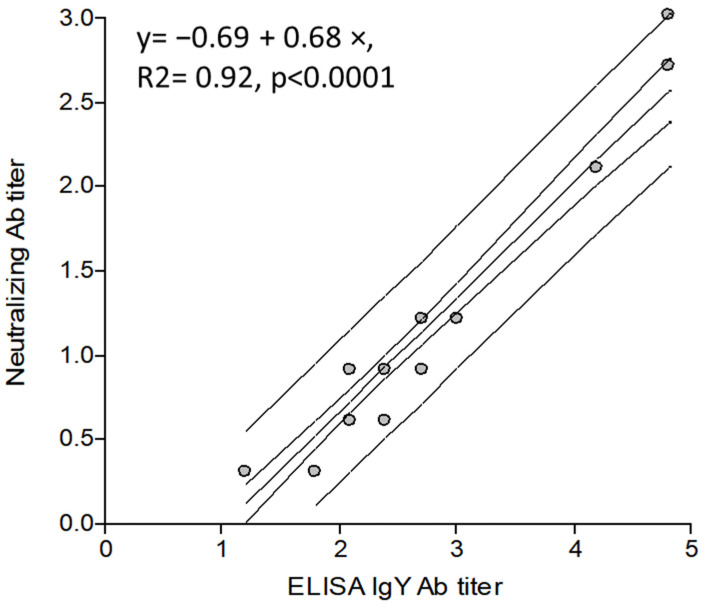
Correlation between ELISA and virus neutralization (VN) Ab titers to determine if the ELISA assay is a versatile alternative tool to measure specific protective Ab titers.

A total of 35 samples (4 hen serum samples, and 31 IgY batches of fluid and spray-dried powder) were tested with ELISA and VN. There was a significant correlation between the Ab titers (R^2^ = 0.92, *p* < 0.001) indicating that the ELISA for IgY quantification might be used as a reference to estimate VN batch-to-batch protective Ab titer (Figure 5).

### 3.4. Reproduction of BCoV Infection and Diarrhea in Colostrum-Deprived Calves

One colostrum-deprived calf was used to produce the virulent BCoV inoculum for further studies. As mentioned in M&M, this calf was infected with 50 mL of BCoV Arg95 strain with a tissue culture infection titer of 10^5^ FFU/mL at 7 days of age. The calf developed diarrhea the day after inoculation. Once BCoV shedding was confirmed, 72 h after virus inoculation, the calf was euthanized and different aliquots of intestinal content were titrated by antigen ELISA, CCIF, and RT-qPCR. All collected fractions were positive for infective BCoV and those titers were much greater than inoculum (Table 4). All fractions were negative for Rotavirus, *E. coli*, and *Salmonella*. A second calf was challenged using 20 mL of the first fecal fraction diluted in D-MEM with antibiotics and filtered by a 0.2 µm syringe filter. This animal received 10^6^ FFU of BCoV which resulted in 6 days of virus shedding and 7 days of diarrhea confirming that the inoculum was able to induce BCoV infection and disease. Microbiological controls and back titration of the inoculum were performed to confirm its identity. Twenty mL of the fecal fraction containing 10^6^ FFU of BCoV was used to challenge calves in the efficacy experiment.

### 3.5. Egg Powder Containing BCoV IgY Abs Reduced Diarrhea Duration and Severity in a Colostrum-Deprived Calf Model

A proof of principle study was conducted to evaluate whether the egg powder enriched in BCoV IgY Abs could prevent BCoV diarrhea in colostrum-deprived calves. As it was detailed in M&M, eight colostrum-deprived calves were randomly assigned to one of the following feeding groups: group 1: calves fed with 2 L of milk supplemented with 40 g of egg powder with a final ELISA BCoV IgY Ab titer of 512 and VN titer of 32, twice a day (*n* = 4) for 14 days; group 2: calves were fed with milk without supplementation (*n* = 4). All animals were challenged orally with 10^6^ FFU of virulent BCoV Arg95 strain (PID 0, 7 days of age). Diarrhea and virus-shedding measurements associated with BCoV infection and disease are summarized in Table 5.

**Figure 6:**
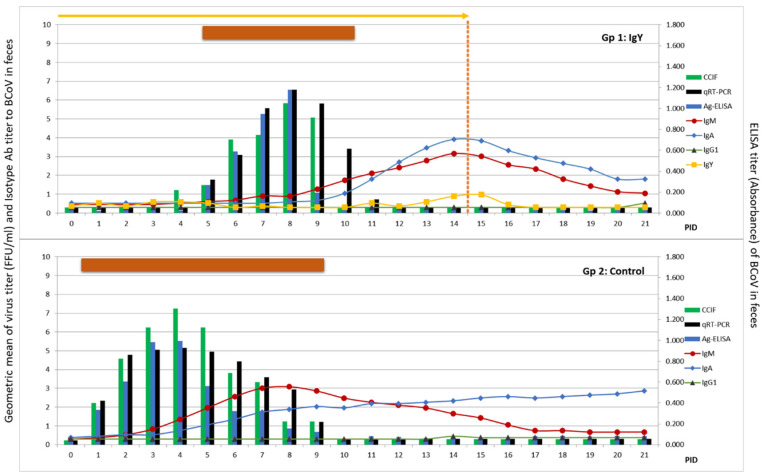
Average of diarrhea evolution in calves from the IgY-treated group (Gp 1, upper panel) and control group (Gp 2, lower panel). Calves from Gp 1 were treated twice a day for 14 days with 40 g of BCoV-specific egg powder in 2 L of milk with a final BCoV ELISA IgY Ab titer of 512 (horizontal arrow). BCoV shedding was measured by CCIF, qRT-PCR, and antigen ELISA expressed in the logarithm of FFU, Cq-value, and Ag ELISA titers expressed in OD from samples diluted 1/10 in PBS. All animals were orally inoculated with 10^6^ FFU of Arg95 strain (0 post-inoculation days (0 PID)) and euthanized at 21 PID. Horizontal bars represent the mean for each group of diarrhea duration (days). BCoV-specific IgM, IgA, IgG1, and IgY titers in fecal samples were expressed in logarithms.

All calves developed diarrhea after inoculation with BCoV. However, calves receiving IgY showed a significant delay (*p* = 0.0019) in the onset of diarrhea (4 days); the diarrhea was significantly less severe (*p* = 0.0036) and significantly shorter in duration (*p* = 0.0003) (50% less duration) than the diarrhea observed in the control group (Table 5, Figure 6). Diarrhea severity (AUC) was also significantly reduced (*p* = 0.0036) in treated calves (Table 5).

All the animals also showed signs of infection and shed BCoV after the challenge. However, the IgY group of calves (Gp 1) showed a significant delay (*p* = 0.0041) in the onset of infectious virus shedding and significantly shorter (*p* = 0.0104) duration (only three days on average) compared with the control group that shed the infectious virus for an average of 6 days (Table 5, Figure 6 and Figure S1). The magnitude of virus shedding (AUC) was reduced compared with the group of animals receiving Ab-free milk, but not significantly (*p*= 0.103) (Table 5).

**Figure 7:**
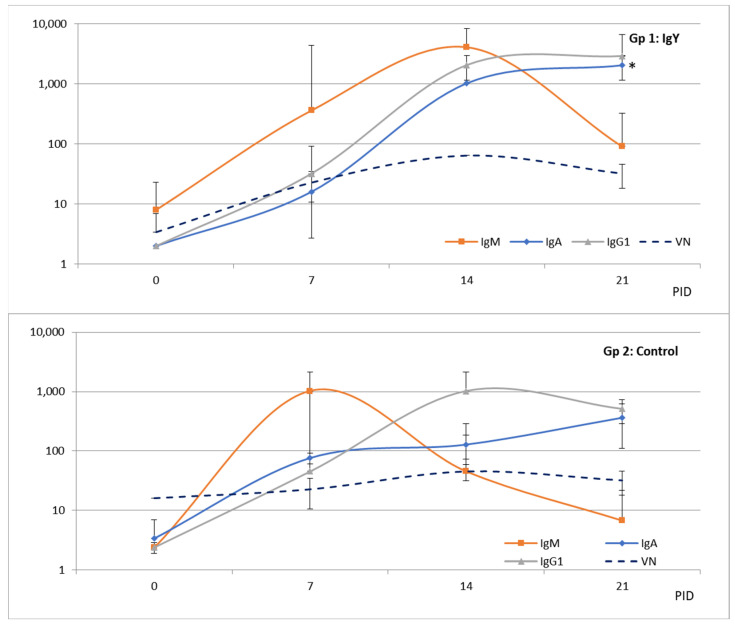
Geometric mean titers (GMT) of BCoV isotype-specific antibody responses in serum from calves in the IgY-treated group (Gp 1, upper panel) and control group (Gp 2, lower panel) at day 0, 7, 14, and 21 post-inoculation (PID). IgM, IgA, and IgG1 were measured by double-sandwich ELISA, and VN antibodies were measured using the Mebus strain. Asterisk means a significant difference (*p* = 0.0210).

When studying the isotype-specific Ab responses to BCoV in feces we observed that fecal-IgM and IgA Ab responses were associated with viral clearance and diarrhea resolution (Figure 6). The peak of IgM responses occurred at 7 PID in the control group compared to 14 PID in the IgY-treated group, in concordance with the delay in the onset of the infection and disease. The peaks of IgA responses were detected in both groups at 14 PID, while higher titers were observed in the calves receiving IgY than in the control animals. No copro-IgG1 Ab or IgG2 responses were detected. In serum, IgA Ab response was higher in the IgY-treated group compared to the control group (*p* = 0.021 at 21 PID) (Figure 7). The peak of IgM Ab response was detected at 7 PID in the serum of the control group compared to 14 PID in the IgY group of calves following the delay of the shedding and disease (Figure 7). IgG1 and VN Ab responses were similar between groups. Seric IgG2 was not detected.

We also evaluated the presence of IgY Ab to BCoV in fecal samples from calves fed with milk supplemented with egg powder enriched in BCoV IgY Abs. We detected IgY Ab in fecal samples of calves from the treated group (Gp 1). As shown in Figure 6, IgY Abs were not detectable in feces during most of the treatment or while shedding was present. IgY Abs to BCoV were detected in calves from Gp 1 between days 10 and 15 once virus clearance was achieved. Even though IgY titers in calves’ feces were low, IgY was specific to BCoV in an ELISA assay, demonstrating that IgY Ab to BCoV is able to travel intact through the gastrointestinal tract to remain detectable until one day after the end of treatment (Figure 6).

Hyperthermia was the last clinical parameter evaluated. Rectal temperature was measured daily in all the calves in this study. Control calves (Gp 2) showed an average of 4.3 days of hyperthermia compared with 1.5 days in calves treated with egg powder enriched in BCoV IgY Ab (Table 5 and Table S1).

## 4. Discussion

There is increasing evidence that all human CoVs currently known might have an animal origin. Controlling BCoV infection in calves is of public health importance since BCoV belongs to the same phylogenetic group as the pandemic SARS-CoV-2 [43]. In addition, very important economic losses are associated with NCD [44] and new strategies are needed to control viral diseases in order to decrease the unnecessary use of antibiotics by farmers. IgY technology is a sustainable source of polyclonal Abs and is aligned with animal welfare because Abs are collected directly from eggs.

In the present study, we describe the development and scaling up of a product based on IgY Ab to control BCoV diarrhea in dairy calves. The ability of IgY to neutralize BCoV infection was measured in vitro. In addition, a binding assay was standardized and statistically validated as a tool to test batch-to-batch consistency.

The binding assay effectively measured IgY Abs to BCoV in serum, fluid egg, and egg powder from BCoV-immunized hens, in feeding milk, and in fecal samples from calves fed with IgY powder. The statistical validation of the assay included the estimation of diagnostic sensitivity and specificity of 97.66% and 98.23%, respectively. During the validation process, five batches of capture polyclonal antibodies, three BCoV antigen batches, and one control set were used. An intermediate precision value of 27% demonstrates the robustness of the assay [37]. As expected, the VN IgY Ab titers to BCoV were lower than ELISA titers. However, there was a significant correlation between the assays. It is important to highlight that even though these VN titers were measured against the Mebus strain and calves were infected with the Arg95 strain, we had already established that Mebus and the local Arg95 strain belonged to the same serotype [12,14].

Although the VN assay might be more precise when detecting antibodies with an effector function, there are many reasons for developing a binding assay to measure IgY antibodies in an egg-based product. For example, egg samples are usually toxic to cell monolayers, and neutralization assays depend on maintaining the integrity of the cell culture. Neutralization assays are also laborious, time-consuming, and need an expert technician to run, read, and interpret the assay. On the other hand, a binding (ELISA) is by far more accessible to most laboratories, gives an objective result, is fast, easy to perform, and has low equipment requirements. Additionally, to confirm a correlation between VN titers and ELISA BCoV-specific IgY titers we performed a regression analysis with log−10 transformed titers resulting in an R^2^ value of 0.92 for a linear regression analysis and 0.97 for the quadratic regression analysis (Supplementary File S1). These results are very optimistic compared to similar studies performed with a correlation analysis between GenScript SARS-CoV-2 ELISA Ab titers and pseudo-VN titers with an R^2^ value of 0.552 [45]. This is the first BCoV-specific IgY ELISA assay to be statistically validated. The assay was transferred to Bioinnovo, a biotechnology firm developing IgY products, and to the National Health Service (SENASA), the local regulatory authority, to control future commercial batches.

The efficacy of the product was tested in a pilot study using a colostrum-deprived neonatal calf model of BCoV infection and disease, specifically standardized for this purpose. Milk supplemented with egg powder enriched in IgY Ab to BCoV was administered to four colostrum-deprived calves and then challenged with 10^6^ FFU of BCoV [18,46,47]. BCoV Arg95 strain was used as the challenge pool because it is a local isolate that genetically represents the strains currently circulating in the field in Argentina and Uruguay [5,12]. We observed that all control calves infected with BCoV showed viral shedding and developed diarrhea for 7.8 days on average starting at 48 h post-inoculation, which was similar to that shown in other studies [18,46,47]. Calves fed with IgY Abs to BCoV with a final ELISA Ab titer of 512 and VN titer of 32 for 14 days showed a significant delay in the onset of diarrhea and virus shedding in contrast to control animals. Moreover, the clinical episodes in the IgY-treated calves were of significantly shorter duration and severity, as was the duration of hyperthermia. This shedding and severity of disease results observed after administering this amount of passive IgY Ab treatment (40 g of egg powder in 2 L of milk, twice a day for 14 days with a final VN titer of 32) are comparable to Ikemori et al.’s results [36]. Ikemori challenged colostrum-deprived calves with 10^9^ TCID50 of Kakegawa BCoV strain and after 6 h calves received 0.5 g of powder egg yolk with a neutralizing titer of IgY Ab to BCoV of 5120 for 7 days, which is equivalent to IgY neutralizing Ab titer in the milk of 12.8 [36]. Other studies performed to evaluate non-specific Ab as passive immunity against BCoV diarrhea show that calves treated with a milk replacement supplemented with commercial serum proteins (APC Lifeline with 10% immunoglobulins, 40% proteins, and 0.5% fat) resulted in no differences in diarrhea severity between the control and treated groups [48]. Finally, several researchers are studying IgY technology against CoV, but they did not evaluate the product in animal models [35,49].

Another aspect to consider is the dose of product administered in each meal. In this study, 40 g in each 2 L milk feeding was the maximum amount of powder that was properly dissolved in the milk. Increasing the Ab titer in the egg powder batches will increase the titer administered in each dose, thus increasing the efficacy of the product to protect against BCoV diarrhea. This could be achieved by optimizing the vaccine formulation containing not only a higher titer of the Mebus reference strain (that grows in proper titers in tissue culture) but also a BCoV strain more closely related genetically to the ones circulating in the field in Argentina, Uruguay, and Brazil [5,50].

It is important to highlight that the efficacy of the product was tested in colostrum-deprived calves representing the worst-case scenario compared to natural conditions with probably lower infectious doses. In previous studies, we observed that calves with BCoV IgG1 passive maternal Abs titers in serum of ≥1024 were mostly protected from BCoV infection compared to calves receiving BCoV IgG1 passive maternal Abs with titers of ≤ 256 [8]. Thus, vaccination of dams with BCoV vaccines followed by proper colostrum intake might result in an optimal level of passive maternal antibody (≥1024). The supplementation of milk with IgY during the susceptible immunological window also represents a double strategy to minimize damage by FPT (colostrum and IgY intake/treatment).

Additionally, we observed that BCoV-specific copro IgM and IgA Ab responses were delayed in the IgY-treated group compared to the control animals. This was correlated with the delay in the onset of diarrhea and virus shedding. Similarly, the serum IgM, IgA, and IgG1 responses were delayed and had a higher magnitude in the treated group compared to the control group. This immunomodulation was also observed in calves experimentally infected with bovine RVA and treated with rotavirus-specific IgY [25] as well as in mice treated with some components of egg yolk [51]. It has been demonstrated that low molecular mass substances associated with chicken IgY stimulated the production of cytokines and nitric oxide from macrophages [52]. Furthermore, the clearance of viral infection and resolution of clinical symptoms was associated with the development of a mucosal immune response characterized by high titers of secretory IgA in the gut.

IgY technology has been successfully used for the development of many potential therapeutics [53] and many of them are being tested in human clinical trials [54]. The development of an IgY-based product to prevent and treat BCoV diarrhea in calves is, to our knowledge, an advance in the field. Although BCoV has been detected with low prevalence in Argentina, BCoV causes diarrhea that complicates clinical symptoms and sometimes leads to the death of the calf [2]. Vaccination is not 100% effective in local dairy production systems, so additional measures might need to be implemented to prevent neonatal diarrhea caused by BCoV. Even if immunization is properly implemented with potency-tested vaccines, colostrum management is still problematic, and calves might succumb to diarrhea. The development of this product might contribute to minimizing the passive immunity shortage and positively modulate gut mucosal immune responses that will protect newborn-treated calves from future exposures to the antigen [25]. Overall, the passive oral administration of BCoV IgY Abs might help reduce the antigen load in farms reducing the risk of diarrhea, while providing a tool to control this viral disease for which antibiotic therapy is not applicable. In addition, a product can be designed to include other pathogens that cause NCD like RVA, *E. coli*, *Salmonella*, etc., to control the syndrome more efficiently or prevent new infections. To our knowledge, this is the first study describing the efficacy of scaled-up DS batches of a spray-dried product based on IgY Abs to BCoV in the prevention of NCD caused by BCoV under controlled conditions.

## 5. Conclusions

Egg powder enriched in IgY to BCoV was successfully developed and produced at an industrial scale obtaining a product to be used in NCD prevention. BCoV infection was reproduced in controlled conditions using a local circulating strain isolated in a dairy farm in Argentina in 1995. Finally, the administration of IgY Ab to BCoV at a final ELISA Ab titer in the milk of 512 and VN Ab titer of 32 as passive treatment significantly delayed the onset and duration of virus shedding and diarrhea in colostrum-deprived calves experimentally challenged with a field strain. Finally, an ELISA assay was developed and statistically validated to control batch-to-batch potency showing high sensitivity and specificity. This study sets the stage to provide an efficient tool to control BCoV diarrhea in dairy farms.

## Figures and Tables

**Table 1:** Binding and neutralizing antibodies (Ab) titers in DS batches of spray-dried egg powder enriched in IgY to bovine coronavirus (BCoV) and in supplemented milk (40 g of spray-dried egg powder/2 Liters of milk).

Product	DS Batch	ELISA	VN (Mebus)	VN (Arg95)	Humidity ^1^ (%)	Fat ^1^(%)	Proteins ^1^ (%)	Coliforms ^2^ (CFU/g)	Salmonella ^2^ (P-A/25 g)
IgY to BCoV	1	1024	64	64	2.97	39.63	48.31	<10	A
IgY to BCoV	2	1024	64	64	3.85	40.61	47.06	<10	A
supplemented Milk		512	32	32	-	-	-	-	-

^1^ Analysis conducted at INTA Institute of Food Technology. ^2^ The presence of contamination with coliforms and Salmonella was measured to ensure the safety of the product. CFU = Colony Forming Units; P = presence; A = Absence, Microbiology lab, INTA Institute of Food Technology.

**Table 2:** ELISA assay statistical validation parameters.

Parameter	Result		Acceptance Criteria
Accuracy and precision measurements	IgY in avian and bovine samples		
Associated parameters	ODc _405 nm_	P%	
Threshold (cut-off)	>0.110	18.68%	ODc = 0.100 P% 13–20%
Diagnosis Specificity (DSp)	98.2%		>90%
Diagnosis Sensitivity (DSe)	97.7%		>90%
Intermediate precision Coefficient of Variation (CV)	27% (Figure 3B)		<30%
Linearity	YES R^2^ = 0.91 *p* = 0.0001		Linear relationship between OD and sample dilution
Reference standard reagent	Sera from chicken immunized with experimental vaccines with known BCoV antigen concentration.		
Operating range of assay	Starting sample dilution 1:32 Linearity was demonstrated between 1024–65536 reciprocal dilutions (Figure 3C)		
“Specificity”	The assay detects IgY antibodies against BCoV but not to Rotavirus, *Salmonella* or *Escherichia coli*		No cross reaction against NCD vaccine antigens

**Table 3:** Concordance analysis between the reference population and the results of the IgY ELISA assay using a cut-off value of 18.68 P% and samples at 1/256 dilution.

	IgY ELISA (Cut-Off: 18.68 P%) Sample Dilution 1/256		
Reference Population	Negative 0	Positive 1	
Negative (0)	108	5	113 (46.9%)
Positive (1)	3	125	128 (53.1%)
	111 (46.1%)	130 (53.9%)	241
Kappa	0.933		
Standard error	0.023		
95% CI	0.888 to 0.979		

**Table 4:** BCoV titers in intestinal contents from a calf inoculated with tissue culture-adapted Arg95 BCoV strain.

Sample	BCoV Infectious Titer (FFU/mL)
Inoculum	10^5^
SIC	10^6^
Colon	10^9^
LIC	10^10^
Feces	10^10^

SIC: Small Intestinal Content; LIC: Large Intestinal Content; FFU/mL: fluorescent focus forming units per milliliter.

**Table 5:** Diarrhea and virus shedding parameters in colostrum-deprived calves fed 2 L of milk supplemented with 40 g of egg powder twice a day for 14 days (group 1: BCoV IgY) or Ab-free milk (group 2: control) experimentally infected with 10^6^ FFU of BCoV Arg95.

		Diarrhea				Shedding of Infectious Virus		
Treatment Group	*n*	Onset (PID)	Duration (Days)	Severity (AUC)	Hyperthermia (Days)	Onset (PID)	Duration (Days)	Magnitude (AUC)
Gp 1: BCoV-IgY	4	6.25 ^A^	3.8 ^B^	17.7 ^B^	1.5 ^B^	6.3 ^A^	3.0 ^B^	23.8 ^A^
Gp 2: Control	4	1.5 ^B^	7.8 ^A^	25.4 ^A^	4.75 ^A^	2.0 ^B^	6.0 ^A^	36.4 ^A^
*p*-value		0.0019	0.0003	0.0036	-	0.0041	0.0104	0.103

Diarrhea severity was measured as the area under the curve (AUC) of calves’ fecal scores (0 to 3 being 2 and 3 considered diarrhea) during 14 days of treatment. The magnitude of virus shedding was measured as the AUC of infectious virus titer by CCIF (log10-transformed). PID: post-inoculation days. Mean in the same column with different superscript letters (A and B) differ significantly (Student *t* test, for all the parameters except for AUC of virus shedding tested by Wilcoxon rank sum test).

## Data Availability

No extra data available.

## Supplementary Materials

The following supporting information can be downloaded at: https://www.mdpi.com/article/10.3390/v15030708/s1, File S1: Regression analysis from ELISA vs VN Ab titers; Figure S1: Diarrhea evolution in each calf from the IgY-treated group and control group. Table S1: Fecal scores and rectal temperatures from calves.
