# Supplementary material for: Development of an IgY-Based Treatment to Control Bovine Coronavirus Diarrhea in Dairy Calves
Source: Viruses. 2023 Mar 9;15(3):708. doi: 10.3390/v15030708 (PMC10059803; doi:10.3390/v15030708)
Supplement: Supplementary file 1 [file viruses-15-00708-s001.zip › viruses-2234981-supplementary.pdf]

File S1. Regression analysis from ELISA vs VN Ab titers.

SAMPLE	ELISA	VN	LOG10_VN	LOG10_ELISA	RDUO LOG10_VN	PRED LOG10_VN
**1**	65536	512	2.71	4.82	0.13	2.57
**2**	16384	128	2.11	4.21	-0.06	2.17
**3**	65536	512	2.71	4.82	0.13	2.57
**4**	65536	1024	3.01	4.82	0.44	2.57
**BATCH 1**	16	2	0.3	1.2	0.18	0.12
**BATCH 2**	16	2	0.3	1.2	0.18	0.12
**BATCH 3**	16	2	0.3	1.2	0.18	0.12
**BATCH 4**	16	2	0.3	1.2	0.18	0.12
**BATCH 5**	16	2	0.3	1.2	0.18	0.12
**BATCH 6**	16	2	0.3	1.2	0.18	0.12
**BATCH 7**	64	2	0.3	1.81	-0.23	0.53
**BATCH 8**	64	2	0.3	1.81	-0.23	0.53
**BATCH 9**	256	4	0.6	2.41	-0.34	0.94
**BATCH 10**	256	4	0.6	2.41	-0.34	0.94
**BATCH 11**	1024	16	1.2	3.01	-0.14	1.35
**BATCH 12**	1024	16	1.2	3.01	-0.14	1.35
**BATCH 13**	1024	16	1.2	3.01	-0.14	1.35
**30**	512	8	0.9	2.71	-0.24	1.14
**31**	512	8	0.9	2.71	-0.24	1.14
**35**	256	4	0.6	2.41	-0.34	0.94
**36**	128	8	0.9	2.11	0.17	0.74
**37**	128	8	0.9	2.11	0.17	0.74
**38**	128	8	0.9	2.11	0.17	0.74
**39**	128	8	0.9	2.11	0.17	0.74
**40**	128	8	0.9	2.11	0.17	0.74
**41**	128	8	0.9	2.11	0.17	0.74
**42**	128	8	0.9	2.11	0.17	0.74
**43**	128	8	0.9	2.11	0.17	0.74
**44**	512	16	1.2	2.71	0.06	1.14
**45**	128	4	0.6	2.11	-0.13	0.74
**46**	128	4	0.6	2.11	-0.13	0.74
**47**	128	4	0.6	2.11	-0.13	0.74
**48**	128	4	0.6	2.11	-0.13	0.74
**49**	128	4	0.6	2.11	-0.13	0.74
**50**	256	8	0.9	2.41	-0.04	0.94

Linear regression analysis

Variable	N	R²	R² Aj	ECMP	AIC	BIC
LOG10_VN	35	0.92	0.91	0.05	-8.18	-3.52

Regression coefficients and associated statistics

Coef	Est.	E.E.	LI(95%)	LS(95%)	T	p-valor
const	-0.69	0.09	-0.88	-0.51	-7.55	<0.0001
LOG10_ELISA	0.68	0.04	0.61	0.75	19.04	<0.0001

Variance Analysis (SC type III)

F.V.	SC	gl	CM	F	p-valor
Model	15.01	1	15.01	362.52	<0.0001
LOG10_ELISA	15.01	1	15.01	362.52	<0.0001
Error	1.37	33	0.04		
Total	16.38	34			

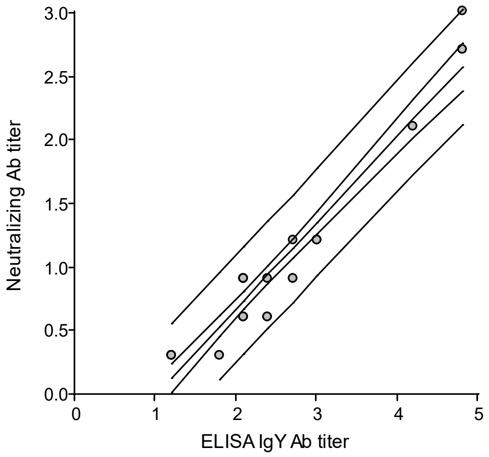

Shapiro-Wilks (modified)

Variable	n	Media	D.E.	W*	p(Unilateral D)
LOG10_VN	35	0.93	0.69	0.74	<0.0001
RDUO_LOG10_VN	35	0.00	0.20	0.86	0.0003

Correlation coeficients

Spearman Correlation: Coefficient/probabilities

	LOG10_VN	LOG10_ELISA
LOG10_VN	1.00	1.1E-11
LOG10_ELISA	0.87	1.00

Base 2 transformation

Linear Regression Analysis

Variable	N	R^2	R^2 Aj	ECMP	AIC	BIC
LOG2_ELISA	35	0.92	0.91	1.03	99.96	104.63

Regression coefficients and associated statistics

Coef	Est.	E.E.	LI(95%)	LS(95%)	T	p-valor
const	3.77	0.27	3.22	4.33	13.88	<0.0001
LOG2_VN	1.35	0.07	1.21	1.50	19.04	<0.0001

Variance Analysis (SC type III)

F.V.	SC	gl	CM	F	p-valor
Model	329.86	1	329.86	362.52	<0.0001
LOG2_VN	329.86	1	329.86	362.52	<0.0001
Error	30.03	33	0.91		
Total	359.89	34			

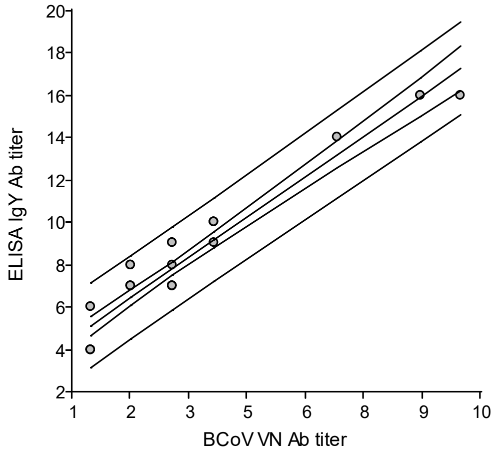

Shapiro-Wilks (modified)

Variable	n	Media	D.E.	W*	p(Unilateral D)
LOG2_ELISA	35	7.94	3.25	0.81	<0.0001
RDUO_LOG2_ELISA	35	0.00	0.94	0.85	<0.0001

No normality in residues

Correlation Coefficients

Spearman Correlation: Coefficient/probabilities

	LOG2_VN	LOG2_ELISA
LOG2_VN	1.00	1.1E-11
LOG2_ELISA	0.87	1.00

Second-grade linearity is tested

Linear Regression analysis

Variable	N	R^2	R^2 Aj	ECMP	AIC	BIC
LOG2_VN	35	0.95	0.95	0.34	61.05	67.27

Regression coefficients and associated statistics

Coef	Est.	E.E.	LI(95%)	LS(95%)	T	p-valor
const	0.27	0.63	-1.01	1.55	0.43	0.6695
LOG2_ELISA	0.07	0.14	-0.21	0.36	0.52	0.6072
LOG2_ELISA^2	0.03	0.01	0.02	0.04	4.44	0.0001

Variance Analysis (SC tipo I)

F.V.	SC	gl	CM	F	p-valor
Model	171.41	2	85.71	294.01	<0.0001
LOG2_ELISA	165.66	1	165.66	568.29	<0.0001
LOG2_ELISA^2	5.75	1	5.75	19.73	0.0001
Error	9.33	32	0.29		
Total	180.74	34			

Variance Analysis (SC type III)

F.V.	SC	gl	CM	F	p-valor
Model	171.41	2	85.71	294.01	<0.0001
LOG2_ELISA	171.41	2	85.71	294.01	<0.0001
Error	9.33	32	0.29		
Total	180.74	34			

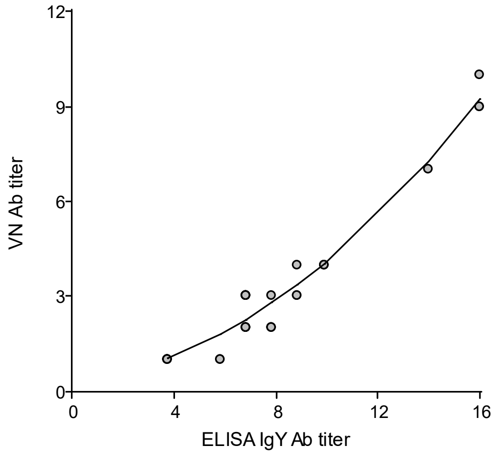

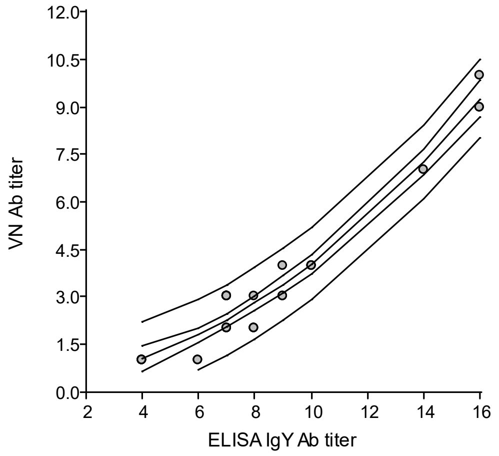

0.27+log2ELISA 0.07 + log2ELISA^2 = log2 VN
R2=0.95, p<0.0001

Shapiro-Wilks (modified)

Variable	n	Media	D.E.	W*	p(Unilateral D)
LOG2_VN	35	3.09	2.31	0.74	<0.0001
LOG2_ELISA	35	7.94	3.25	0.81	<0.0001
RDUO_LOG2_ELISA	35	0.00	0.94	0.85	<0.0001

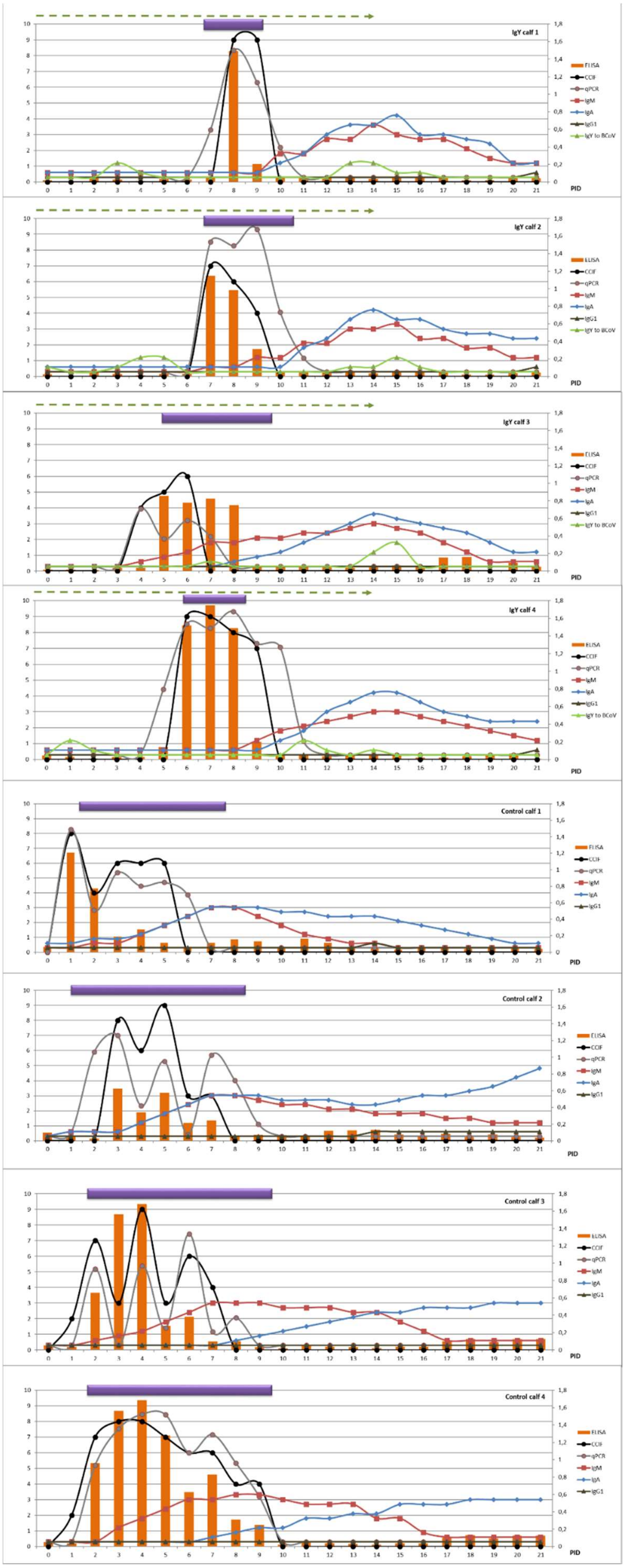
IgY calf 1
ELISA
CCIF
qPCR
IgM
IgA
IgG1
IgY to BCoV
PID
IgY calf 2
IgY calf 3
IgY calf 4
Control calf 1
Control calf 2
Control calf 3
Control calf 4

Figure S1. Diarrhea evolution in each calf from the IgY-treated group and control group. Calves from Gp 1 were treated twice a day for 14 days with 40 g of BCoV-specific egg powder in 2 L of milk with a final BCoV ELISA IgY Ab titer of 512 (horizontal arrow). BCoV shedding was measured by CCIF, qRT-PCR, and antigen ELISA expressed in the logarithm of FFU, Cq-value, and Ag ELISA titers expressed in OD from samples diluted 1/10 in PBS. All animals were orally inoculated with 10^6 FFU of Arg95 strain (0 post-inoculation days (0 PID)) and euthanized at 21 PID. Horizontal bars represent the mean for each group of diarrhea duration (days). BCoV-specific IgM, IgA, IgG1, and IgY titers in fecal samples were expressed in logarithms.

Table S1. Fecal Scores and rectal temperature. Fecal consistency was scored from 0 to 3 every day from 0 DPI to 21 PID (0: normal; 1: pasty; 2: semi-liquid; 3: liquid). A score equal to or greater than 2 was considered diarrhea. Rectal temperature was measured every day from 0 DPI to 21 PID. Temperature higher than 39.4°C was considered hyperthermia.

Group	Score				Rectal Temperature				Group	Score				Rectal Temperature			
	C1	C2	C3	C4	C1	C2	C3	C4		C1	C2	C3	C4	C1	C2	C3	C4
	1	0	0	0	39.4	39.1	38.5	38.8		0	1	1	1	39.2	38.6	38.6	38.8
	3	2	3	0	39.2	38.9	39.5	39.3		0	1	0	1	39.3	39.1	38.8	38.7
	3	2	2	3	39.0	40.6	40.0	39.6		1	1	1	1	38.9	40.0	39.1	38.8
	3	2	3	3	39.0	38.8	39.7	40.3		1	1	1	1	38.8	38.9	39.0	39.0
	3	3	3	3	40.3	41.1	39.1	39.4		1	1	1	1	39.4	39.3	39.3	39.1
	3	3	3	3	40.3	39.4	39.9	41.0		3	1	1	1	40.5	39.1	39.8	38.9
	3	3	2	3	38.9	39.9	39.7	39.3		3	2	1	1	39.1	38.0	39.4	39.8
	2	3	2	3	40.6	39.9	39.9	39.6		3	2	2	2	39.0	39.3	39.9	40.1
	1	2	2	2	39.6	40.7	39.3	39.2		3	2	2	3	38.8	38.8	39.2	39.4
	1	1	1	2	39.4	39.4	38.8	39.4		3	1	2	2	38.8	39.0	39.1	39.2
Control	1	1	1	1	39.4	39.3	39.2	38.9	IgY	1	0	2	1	38.4	38.9	38.8	39.2
	1	1	1	1	39.3	39.0	39.0	39.0		1	1	1	1	39.3	39.2	38.9	38.8
	1	1	1	1	39.3	38.8	38.8	39.2		1	1	1	1	39.2	38.7	39.0	38.9
	1	1	1	0	39.4	38.9	39.4	38.9		1	1	1	1	39.4	38.6	39.1	38.7
	1	1	1	0	39.2	38.8	39.4	38.8		1	1	1	0	39.1	39.0	38.8	38.8
	0	1	1	1	39.1	39.3	39.2	39.2		1	0	1	1	39.0	39.1	39.2	39.0
	0	1	1	0	39.0	39.1	38.9	39.0		0	1	1	1	39.4	38.9	38.7	39.2
	1	1	0	0	39.4	39.4	39.3	39.2		0	1	0	0	39.2	38.9	38.8	38.9
	0	1	1	0	39.2	39.4	39.1	39.3		1	1	1	1	39.2	39.0	38.9	38.8
	0	1	1	1	39.2	38.7	39.4	39.3		1	1	1	1	38.9	38.8	38.9	39.0
	0	0	1	1	39.3	38.9	38.8	39.1		1	1	1	1	38.8	38.4	39.0	38.9
	1	0	1	0	39.0	39.0	38.9	39.3		1	1	1	1	39.0	39.0	38.8	39.1
